# Pharmacokinetics of tenofovir alafenamide, emtricitabine, and dolutegravir in a patient on peritoneal dialysis

**DOI:** 10.1186/s12981-024-00616-5

**Published:** 2024-05-21

**Authors:** Sandra Abdul Massih, Mohamed G. Atta, Chloe L. Thio, Jeffrey A. Tornheim, Edward J. Fuchs, Rahul P. Bakshi, Mark A. Marzinke, Craig W. Hendrix, Ethel D. Weld

**Affiliations:** 1 Division of Clinical Pharmacology, Department of Medicine, The Johns Hopkins University School of Medicine, Baltimore, MD USA; 2 Division of Nephrology, Department of Medicine, The Johns Hopkins University School of Medicine, Baltimore, MD USA; 3 Division of Infectious Diseases, Department of Medicine, The Johns Hopkins University School of Medicine Baltimore, Baltimore, MD USA; 4 Department of Pathology, The Johns Hopkins University School of Medicine, Baltimore, USA

**Keywords:** HIV treatment, Peritoneal dialysis, Tenofovir, Emtricitabine, Nephrotoxicity, Trough concentration

## Abstract

**Introduction:**

Peritoneal dialysis (PD) is an effective renal replacement modality in people with HIV (PWH) with end-stage kidney disease (ESKD), particularly those with residual kidney function. Data on pharmacokinetics (PK) of antiretrovirals in patients on peritoneal dialysis are limited.

**Methods:**

A single-participant study was performed on a 49-year-old gentleman with ESKD on PD and controlled HIV on once daily dolutegravir (DTG) 50 mg + tenofovir alafenamide (TAF) 25 mg / emtricitabine (FTC) 200 mg. He underwent serial blood plasma, peripheral blood mononuclear cell, and urine PK measurements over 24 h after an observed DTG + FTC/TAF dose.

**Results:**

Plasma trough (Cmin) concentrations of TAF, tenofovir (TFV), FTC, and DTG were 0.05, 164, 1,006, and 718 ng/mL, respectively. Intracellular trough concentrations of TFV-DP and FTC-TP were 1142 and 11,201 fmol/million cells, respectively. Compared to published mean trough concentrations in PWH with normal kidney function, observed TFV and FTC trough concentrations were 15.5- and 20-fold higher, while intracellular trough concentrations of TFV-DP and FTC-TP were 2.2-fold and 5.4-fold higher, respectively. TFV and FTC urine levels were 20 times lower than in people with normal GFR.

**Conclusions:**

In a single ESKD PWH on PD, daily TAF was associated with plasma TFV and intracellular TFV-DP trough concentrations 15-fold and 2-fold higher than those of people with uncompromised kidney function, potentially contributing to nephrotoxicity. This suggests that TFV accumulates on PD; thus, daily TAF in PD patients may require dose adjustment or regimen change to optimize treatment, minimize toxicity, and preserve residual kidney function.

## Introduction

People living with HIV (PWH) are at a higher risk for developing chronic kidney disease (CKD) than the general population. In North America, up to 1 in 10 individuals living with HIV has CKD, due to both HIV-related factors and traditional risk factors [1–4]. Peritoneal dialysis (PD) is a form of kidney replacement therapy that has been increasing in use globally and in the USA, where up to 10% of people needing dialysis are on PD [5]. However, data on antiretroviral pharmacokinetics (PK) and dosing in this population are scarce. Descovy™ (fixed dose formulation of the nucleoside reverse transcriptase inhibitors emtricitabine (FTC) and tenofovir alafenamide (TAF) lacks an FDA label indication for people with severe kidney disease (creatinine clearance (CrCl) < 30 mL/min) who are not yet on dialysis, but can be used in individuals with CrCl < 15mL/min who are on hemodialysis (HD) without dose adjustment, with recommended dosing timed after HD [6–8]. 

TAF is a modified prodrug of tenofovir (TFV); it is administered at lower dosages than tenofovir disoproxil fumarate (TDF) and is associated with enhanced prodrug stability in plasma and lower systemic TFV exposures. Studies of healthy individuals switched from TDF to TAF showed 90% lower plasma TFV concentrations and 2- to 4-fold higher intracellular TFV-DP concentrations with TAF than with TDF [9]. The lower plasma TFV concentration is largely responsible for the improved kidney and bone toxicity profile of TAF [10]. Studies in individuals with severe CKD (CrCl of 15 to 29 mL/min) given TAF have shown that plasma peak concentration (C_max_) and area under the concentration-time curve extrapolated to infinity (AUC_inf_) of TAF and TFV are 79% and 92% higher, and 2.8-fold and 5.7-fold higher, respectively, than in individuals with normal kidney function given TAF [11].

Conversely, the integrase strand transfer inhibitor (INSTI) dolutegravir (DTG) may be used for people with severe CKD (CrCl < 30 mL/min) who tend to have lower plasma DTG concentrations for unexplained reasons [12], a small case series of the use of daily DTG in people on HD have found it to be safe and effective without dose adjustment [13].

While some scant data on TAF dosing in people with ESKD on HD are available [14, 15], the pharmacokinetics of TAF in people on PD have not been characterized. There is a single case report in the literature of a 46-year-old patient with HIV and HBV on PD who was taking TDF 245 mg once weekly + ritonavir-boosted atazanavir (r/ATZ). Plasma TFV concentrations were measured before and at 2 and 4 h into a peritoneal dialysis session with a 4-hour dwell; observed TFV trough concentrations were 510 ng/mL in serum and 200 ng/mL in the 24-hour dialysis fluid, confirming that TFV is partially extracted by PD. In order to lower concentrations to achieve target steady state concentrations (50–300 ng/mL), TDF dosing was decreased to 245 mg every 2 weeks; post-dose adjustment, observed serum TFV concentrations were 200 ng/mL [16].

To our knowledge, the current report is the first in the literature to describe the PK of TAF in a PWH on peritoneal dialysis.

## Case presentation

A 49-year-old African American gentleman with past recovery from hepatitis B virus (HBV) infection and stably controlled HIV (CD4: 255 cells/mm^3^ (13.5%); HIV RNA: < 20 copies/mL) developed ESKD in the past 2 years due to type 1 diabetes and hypertension (he denied ingesting any nephrotoxins over this period.) He had been initiated one year prior on continuous 4-cycler PD nightly via an abdominal peritoneal dialysis catheter. At the time of PD initiation, he was found to be a low average transporter with the peritoneal equilibration test (PET). He was consented and brought into the Clinical Research Unit for sampling on two consecutive days. Eleven months prior to the study visit he had a hospitalization for bacterial peritonitis related to his PD catheter. At the time of study visit, his eGFR was 6 mL/min/1.73 m^2^ (eGFR CKD-Epi (2021) equation) and he was placed on the transplant list for a kidney-pancreas transplant. His overnight PD was followed by morning dosing of his ART. He had been on a TAF-containing regimen for 5 years and had initiated a regimen of once-daily 50 mg DTG/200 mg FTC/25 mg TAF 7 months prior to the described study visit. He was prompted daily to take his ART for 3 days prior to presenting to the clinical trials unit for pharmacologic sampling; pre-dose blood was collected, followed by his observed standard dose of DTG/FTC/TAF. Blood and urine were then collected over a 24-hour period.

## Methods

The study was conducted at the Johns Hopkins University School of Medicine’s Drug Development Unit under an institutional review board (IRB)-approved protocol (NA_00031939); the participant provided informed consent. The study included a screening visit to determine eligibility based on the participant taking one of the protocol’s approved drugs, followed by the study visit. Blood was collected for plasma and PBMC isolation pre-dose, and 0.25, 0.5, 1, 1.5, 2, 3, 4, 6, 8, 10, and 24 h after the observed dose. Urine was collected cumulatively over two time periods, 0–10 h, and 10–24 h post-dose with urine volume totals recorded. The participant’s plasma trough concentrations for all drugs were compared pre-dose and at 24 h after dose, to assess if he was at steady state on his ART. PK parameters were compared between the person included in this case report and cohorts of PWH, both with normal kidney function and on hemodialysis (HD). (Table 1). The PK parameters compared included time until maximum plasma concentration (T_max_), (C_max_), minimum plasma concentration (C_min_), and (AUC_last_). Renal dose was calculated from the 24-hour cumulative urine volume and the urine drug concentration. Renal clearance was then calculated as described before [17, 18]. KT/V and urea clearance values were calculated in his Nephrology chart with an online calculator where K is urea clearance, T is time on dialysis, and V is the urea volume of distribution where K is urea clearance, T is time on dialysis, and V is the urea volume of distribution [19, 20].

**Table 1:** Plasma and intracellular PK parameters for TAF, TFV, FTC, and DTG from a participant with HIV on PD & Comparison (ratio) of parameters to those in PWH with normal kidney function (normal CrCl)

Drug	Matrix	T_max_ (hours)	C_max_ (ng/mL)	C_max_ Ratio PD/Normal CrCl	AUC_last_ (h*ng/mL)	AUC_last_ Ratio PD/Normal CrCl	C_min_ (ng/mL)	C_min_ Ratio PD/Normal CrCl
_**TAF**_	_**Plasma**_	_0.5_	_311.3_	_1.92_	_289.8_	_1.40_	_0.05_	_−_
_**TFV**_	_**Plasma**_	_4_	_169.4_	_11.14_	_3,905.6_	_13.33_	_164.4_	_15.51_
_**FTC**_	_**Plasma**_	_2_	_2991_	_1.74_	_44,522_	_5.56_	_1,006_	_20.12_
_**DTG**_	_**Plasma**_	_4_	_1719_	_0.51_	_26,401_	_0.60_	_717.8_	_0.86_
_**TFV−DP**_	_**PBMC**_	_5.6_	_1,554.10_	_1.74_	_27,172.61_	_2.04_	_997.91_	_2.18_
_**FTC−TP**_	_**PBMC**_	_5.6_	_21,068.61_	_4.68_	_294,599.8_	_4.17_	_9,789.94_	_5.44_

Data from individuals with normal creatinine clearance based on results from:

a) Two phase III trials (GS-US-292-0104 and GS-US-292-0111) for TAF and TFV [6, 8]

b) Data from Phase III trial FTC-101 for FTC [6, 8]

c) Data from Min et al. for DTG [21]

d) Data from Thurman et al. for TFV-DP and FTC-TP [22]

CrCl = creatinine clearance; TAF = tenofovir alafenamide; FTC = emtricitabine; TFV = tenofovir; DTG = dolutegravir; T_max_ = time of maximal concentration; C_max_ = maximal concentration; AUC_last_ = area under the concentration-time curve from time 0 until the last observed concentration; C_min_ = trough concentration at 24 hours (the end of the dosing interval) PBMC = peripheral blood mononuclear cells; TFV-DP = tenofovir-diphosphate (active intracellular metabolite of tenofovir); FTC-TP = emtricitabine triphosphate (active intracellular metabolite of emtricitabine)

Drug concentrations were determined via liquid chromatographic-mass spectrometric (LC-MS/MS) analysis using previously described methods by the Clinical Pharmacology Analytical Laboratory (CPAL) within the Johns Hopkins University School of Medicine [23–25]. Assay lower limits of quantification (LLOQ) were as follows: plasma TAF, 0.03 ng/mL; plasma TFV: 1 ng/mL; plasma FTC: 5 ng/mL; plasma DTG: 100 ng/mL; urine TFV, 50 ng/mL; urine FTC, 50 ng/mL; PBMC tenofovir diphosphate (TFV-DP), 5 fmol/sample; PBMC emtricitabine triphosphate (FTC-TP), 50 fmol/sample. Intracellular anabolite concentrations were normalized to cell counts and reported as fmol/million cells.

## Results

The participant was initially non-oliguric and continued to produce urine throughout the 24-hour study visit. Dialysis dose delivered was quantified by the KT/V ratio and residual kidney function was quantified by the urea clearance (for reference, normal kidneys clear urea at a rate of 65 mL/min, equating to 655 L of blood per week). KT/V ratio and residual renal urea clearance were 1.82 and 0.58 L/week six weeks before the study visit, 1.84 and 0.07 L/week one month after the study visit, 1.84 and 0.43 L/week 4 months after the study visit, and 1.62 and 0.18 L/week six months after the study visit, respectively [19, 20]. Viral suppression was maintained.

**Fig. 1:**
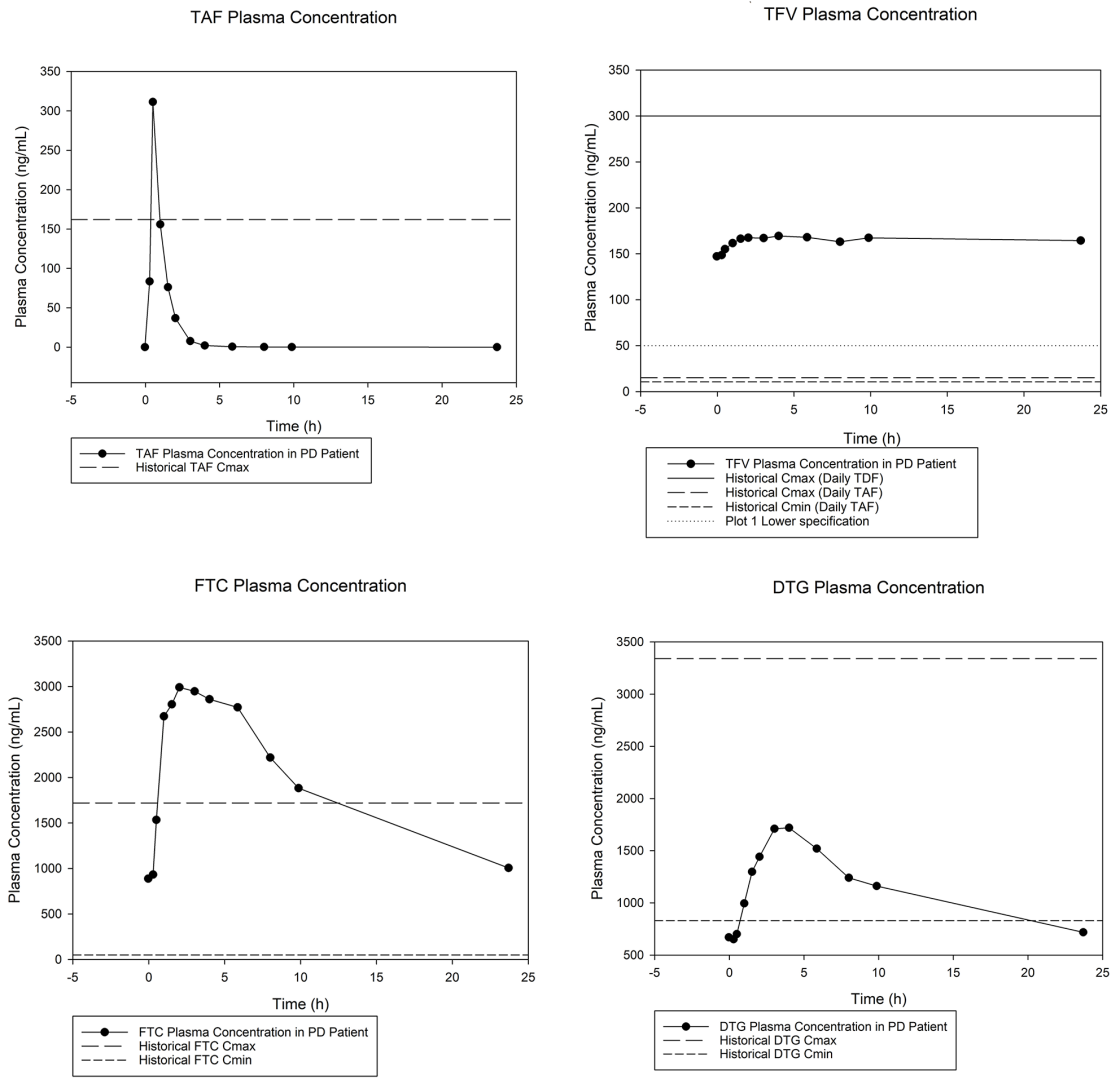
Plasma concentration: time plots of TAF, TFV, FTC, and DTG. Plasma drug concentration versus time plots for each of the four analytes related to the three drugs studied. Dotted reference lines indicate historical C_max_ (long dash) and C_min_ (short dash) for TAF, TFV, FTC, and DTG historical data. TFV plot includes additional historical C_max_ (solid line) and C_min_ (dotted line) from TDF dosing

Plasma concentration time profiles were plotted in relation to PK values in those with normal renal function (Fig. 1). Plasma pre-dose and 24-hour post-dose trough concentrations were 670 and 718 ng/mL for DTG, 147 and 164 ng/mL for TFV, and 888 and 1006 ng/mL for FTC, respectively, indicating that the participant may not have been at steady-state for his ART medications. The TAF C_min_ was below the limits of quantitation of 0.05 ng/mL. When compared with concentrations in PWH with normal kidney function, (Tables 2 and 1) TAF C_max_ and AUC_last_, were 1.92 and 1.40 times higher respectively; elevations were more pronounced for TFV, as C_max_ and AUC_last_ were 11.1 and 13.3-fold higher in the participant undergoing PD than in PWH with normal renal function. FTC C_max_ and AUC_last_ were 1.74 and 5.56-fold higher. C_min_ was 15.5-fold and 20- fold higher for TFV and FTC, respectively. Lastly, DTG C_min,_ C_max_ and AUC_last_ were 0.90-, 0.51-and 0.60-fold lower, respectively.

**Table 2:** Comparison of Plasma and Intracellular PK parameters for TAF, TFV, FTC, and DTG between the participant with HIV on PD and other populations with and without HIV and renal impairment

Drug	Population	Sample size (# individuals)	T_max_ (hours)	C_max_ (ng/mL)	C_max_ Ratio PD/Normal CrCl	AUC_last_ (h*ng/mL)	AUC_last_ Ratio PD/Normal CrCl	C_min_ (ng/mL)	C_min_ Ratio PD/Normal CrCl
**TAF**	**HIV+, Normal CrCL** ^(a)(b)^	*N* = 539	1	162		206			
	**HIV-, Renal impairment** ^(b, c)^	*N* = 13		364 (65.7)		513 (47.3)			
	**HIV+, PD**	1	0.5	311.3	**1.92**	289.8	**1.40**	0.05†	
	**HIV+, HD** ^(b, d)^	*N* = 12		246 (75%)	**1.26‡**	232 (53)	**1.25‡**		
**TFV**	**HIV+, Normal CrCL** ^(a, b, e)^	*N* = 841	1	15.2 (26.1)		293 (27.4)		10.6 (28.5)	
	**HIV-, Renal impairment** ^(b, c)^	*N* = 14		26.4 (32.4)		2,070 (47.1)			
	**HIV+, PD**	*N* = 1	4	169.4	**11.14**	3905.6	**13.33**	164.4	**15.51**
	**HIV+, HD** ^(b, d)^	*N* = 10		443 (41)	**0.38‡**	8,720 (39)	**0.45‡**	265 (73)	**0.6‡**
**FTC**	**HIV+, Normal CrCL** ^(a, b, e)^	*N* = 8	1 (1, 2)	1,720 (53)		8,000 (15)		50 (24)	
	**HIV+, PD**	*N* = 1	2.0167	2,991	**1.74**	44,522.08	**5.56**	1,006	**20.12**
	**HIV+, HD** ^(b, e)^	*N* = 11		4,880 (41)	**0.61‡**	62,900 (48)	**0.71‡**	1280 (59)	**0.79‡**
**DTG**	**HIV+, Normal CrCL** ^(g, b)^	*N* = 10	2	3,340 (16)		43,400 (20)		830 (26)	
	**HIV+, PD**	*N* = 1	4	1,719	**0.51**	26,401.27	**0.60**	717.8	**0.86**
	**HIV+, HD** ^(h)^			1,894	**0.91‡**				
**PBMC TFV-DP**	**HIV-, normal CrCl** ^(i, j)^ Median (range)	*N* = 24	2 (1, 48)	892.35 (388.06, 5,004.52)		13,297.91 (7,603.8, 37,310.19)		457.1 (238.6, 813.69)	
	**HIV + on PD** ^(k)^	*N* = 1	5.6	1,554.10	**1.74**	27,172.61	**2.04**	997.91	**2.18**
**PBMC FTC-TP**	**HIV-, normal CrCl**	*N* = 24	2 (1, 8)	4,500.23 (2,793.30, 23,531.58)		70,695.24 (50,554.78, 151,745.63)		1,800.44 (1,147.562, 3,443.56)	
	**HIV + on PD**	*N* = 1	5.6	21,068.61	**4.68**	294,599.8	**4.17**	9,789.94	**5.44**

HD = hemodialysis; PD = peritoneal dialysis; CrCl = creatinine clearance; TAF = tenofovir alafenamide; FTC = emtricitabine; TFV = tenofovir; DTG = dolutegravir; T_max_ = time of maximal concentration; C_max_ = maximal concentration; AUC_last_ = area under the concentration-time curve from time 0 until the last observed concentration; C_min_ = trough concentration at 24 h (the end of the dosing interval) PBMC = peripheral blood mononuclear cells; TFV-DP = tenofovir-diphosphate (active intracellular metabolite of tenofovir); FTC-TP = emtricitabine triphosphate (active intracellular metabolite of emtricitabine); CrCl = creatinine clearance; PD = peritoneal dialysis

a) Based on data from two pivotal phase III trials (GS-US-292-0104 and GS-US-292-0111). [6, 8]

b) Data represented in Mean (CV%)

c) Based on data by Custodio et al. [11]

d) Based on data by Eron et al. [14, 15]

e) Based on data from Phase III trial FTC-101 [8]

f) Based on data from Min et al. [21]

g) Concentration is average of 5 patients’ levels post hemodialysis. Dialysis was performed ∼ 5.9 h post dose. Data by Molto et al. [13]

h) Based on data by Thurman et al. [22]

i) Data represented in Median (range)

j) eGFR -CKD-EPI Creatinine for patient = 6 mL/min/1.73 m^2^

† C_min_ for TAF was below the limit of quantification for the assay (BLQ < 0.03 ng/mL). This value represents the C_last_ that was detected 10 h

**‡** Ratio of PD/HD data

Intracellular TFV-DP and FTC-TP concentrations were compared with historical and published data (Table 2); TFV-DP C_max_, AUC_last_, and C_min_ were 1.74 times, 2.04 times, and 2.18 times higher in the participant receiving PD (Table 2). TFV-DP C_max_ and AUC_last_ were still within the range of concentrations observed in those with non-compromised renal function. For FTC-TP, C_max_ was 4.68 times higher but within normal range, while AUC_last_, and C_min_ were 4.17 and 5.44 times higher, respectively, and out of range when comparing his measurements with the median FTC-TP exposures of people with normal kidney function.

The participant produced 615 mL of urine over a 24-hour period. Total urine concentrations for FTC and TFV were 56,380 ng/mL and 6,743 ng/mL, respectively, for the first (0–10 h) period, and 27,970 ng/mL and 4,524 ng/mL, respectively, for the 10-24-hour period. Dose and renal clearance were calculated for both drugs. For TFV, the cumulative amount excreted (A_0 − 24_) was 3.3 mg, which makes up 22% of the 15 mg of TFV provided by 25 mg of TAF [26]. The TFV renal clearance was 14.1 mL/min. For FTC, (A_0 − 24_) was 23.86 mg, which is 11.9% of total 200 mg dose, with renal clearance 8.93 mL/min.

## Discussion

We present the first report on TAF PK in a person with HIV with ESKD on chronic PD. Both C_max_ and AUC of TAF in this participant were comparable with TAF concentrations in individuals with normal kidney function, likely due to the fact that TAF is not renally cleared to a significant degree [11, 27]. However, plasma TFV concentrations were higher in the setting of PD, ranging from 11-fold (C_max_) to 15-fold (C_min_) higher compared to individuals with normal kidney function. The elevated TFV trough observed in the participant on TAF in the setting of PD likely indicates plasma accumulation. Notably, the TFV trough concentration was also 3-fold higher than what would be expected with steady-state TDF dosing in someone with normal renal function (median trough concentration of ∼ 50 ng/mL (IQR 35–77) [28–30]. 

While FTC C_max_ was modestly higher in our PD patient compared to patients with normal renal function, both FTC AUC_last_ and FTC trough (C_min_) were many-fold higher—6-fold and 20-fold higher, respectively. This suggests FTC accumulation in the plasma, however, this may not add substantial toxicity risk given the overall tolerability of FTC [31]. Lastly, DTG peak, trough, and AUC_last_, measurements were lower in this participant than in people with normal kidney function. This might indicate that DTG is either (1) better cleared by PD (compared to HD where it is only minimally cleared, with a median extraction ratio of 7%) or (2) not being absorbed as well, or (3) another mechanism that is not yet characterized [13]. Regardless, the DTG trough concentrations, while low, are above the protein-adjusted in vitro IC90 of 64 ng/mL and also above 300 ng/mL, the median plasma trough concentrations established to be sufficient for viral suppression from 10 mg DTG once daily,, which showed equivalent viral suppression to recommended 50 mg once daily in the phase 2 efficacy trial SPRING-1) [32].

Despite the high plasma TFV concentrations, TFV-DP C_max_ and AUC _last_ were within the normal range, while C_min_ was slightly above the range, 2.18 times the average historical data. For FTC-TP, C_max_ was within range, while AUC_last_ and C_min_ were higher compared with historic data, with C_min_ being 5.44-fold higher than the historical average. The molar relationship between plasma FTC and intracellular FTC-TP (0.1) is higher than previously reported (0.034) [33, 34, 22] and may be attributed to the 20-fold higher plasma FTC trough concentrations, or the saturation of one of the molecular mechanisms responsible for the conversion of FTC to FTC-TP [8, 35, 36]. 

Comparing the participant’s urine data to a recent study of people with normal renal function taking FTC/TDF [37], the TFV A_0 − 24_ was 38 mg, which made up 28% of the 136 mg TFV provided by 300 mg TDF, while the clearance was 289 mL/min, which is 20-fold higher than the clearance in the participant. This supports that TFV is not getting sufficiently cleared in the participant by his kidneys nor by his PD, causing the accumulation. As for FTC, in those with normal renal function, the A_0 − 24_ was 114 mg, making up 57% of the 200 mg dose of the FTC. Clearance was 216 mL/min, 24-fold the clearance in the participant.

The factors that determine whether a given drug is likely to be removed via PD include drug specific factors like molecular weight, protein binding, water solubility, and volume of distribution, as well as patient specific factors like their peritoneal membrane transport function [38, 39]. TFV it is cleared by HD, with an extraction ratio of around 54%, and has factors that suggest it should be easily dialyzable via PD [14, 40]. However, since plasma TFV concentrations were quite elevated in this participant on PD, the amount of TFV removed with PD is likely insufficient to overcome the accumulation that occurs in the absence of renal elimination. And although the TFV concentrations were lower compared with historical HD data, those were from people dosed with TDF. Bloodstream TFV concentrations in the setting of daily adherence to TAF might be even higher, given that this individual was not at steady state. Further, the TFV accumulation observed in our PD patient led to TFV plasma trough concentration threefold those seen with daily TDF dosing, possibly mitigating any renal safety advantages conferred by TAF compared to TDF, as concentrations in this range have previously been linked to potential nephrotoxicity [41, 42]. 

Between the time of the study visit and the publication of this report, the patient’s residual kidney function had declined further, fluctuating around a low baseline. We do not know if this resulted from continued progression of his underlying renal disease or elevated plasma TFV concentrations. Regardless, based on data from this single individual on PD, we conclude that once daily TAF dosing results in plasma trough TFV concentrations 15-fold higher than those in individuals with normal kidney function, and 3-fold higher than trough TFV concentrations in individuals with normal kidney function on TDF. Notably, we do not judge any of the observed PK changes in the participant on PD to have resulted in any loss of antiviral efficacy; he has remained suppressed. But while more research is needed, it may be reasonable at present to avoid daily TAF in people with ESKD receiving PD where preservation of residual kidney function is strongly desired.

## Data Availability

The deidentified data that support the findings of this study are available on request from the corresponding author. The data are not publicly available due to privacy or ethical restrictions.
